# Triggering change in diabetes care delivery in general practice: a qualitative evaluation approach using the clinical microsystem framework

**DOI:** 10.1186/1471-2296-15-32

**Published:** 2014-02-13

**Authors:** Tina Janamian, Lisa J Crossland, Claire Jackson, Jenny Morcom

**Affiliations:** 1 Centre for Research Excellence in Primary Health Care Microsystems, University of Queensland, Level 8, Health Sciences Building, Building 16/910, Royal Brisbane Hospital, Herston, Brisbane, Queensland, 4006, Australia; 2 Focus Health Network, 7 The Esplanade Cotton Tree, Maroochydore, Queensland, Australia

**Keywords:** Clinical microsystems, Quality improvement, Diabetes mellitus, Chronic disease management, Health services research

## Abstract

**Background:**

In 2008, the Sunshine Coast Division of General Practice (SCDGP) in Queensland, Australia initiated a highly successful Improved Diabetes Management (IDM) program with general practices in a regional area. The IDM program was evaluated against the 10 elements of a high functioning clinical microsystem framework as identified by Nelson et al. (2007) in order to determine key factors contributing to the successful adoption and uptake of the program in participating general practices.

**Methods:**

The evaluation focussed on in-depth key informant interviews with 10 SCDGP staff and general practitioners (GPs) involved in the IDM program. A thematic analysis was undertaken and common emergent themes were reviewed against the 10 elements of high performing clinical microsystem.

**Results:**

While all aspects of the clinical microsystem approach appeared effective in the design, implementation and adoption of the IDM program, several characteristics were crucial. The identification of champions of change in both the division and participating practices, the celebration of positive achievements and the use ‘real data’ from practices to demonstrate improved health outcomes for patients from the practice were instrumental in motivating participating GPs to both implement and sustain changes in their diabetes care delivery.

**Conclusion:**

In designing and redesigning health care, the clinical microsystems approach offers a pathway for the effective uptake of innovation in Australian primary health care; a means of integrating structure, process and outcomes of a care framework for reviewing improvements in the health care delivery process and could lead to improvements in patient health outcomes.

## Background

The Australian Divisions of General Practice aim to guide and support improvements to health care delivery in general practices within a defined geographic area. Currently general practitioners (GPs) screen for and manage the majority of diabetes complications and achieving best practice diabetes care is a goal of many of these organisations. In 2008, the Sunshine Coast Division of General Practice (SCDGP) on the east coast of Queensland, Australia initiated and led an Improved Diabetes Management (IDM) program with 12 general practices. The design and implementation of this innovative program was based on the clinical microsystem approach. This approach focuses on the microsystems in health care, defined as “the small, functional, front-line units that provide most health care to most people” and are “essential building blocks of larger organisations and of the health system” [1]. Although change is required at all levels of the system, the powerful microsystem concept offers an opportunity to understand and transform health care at the front-line of service delivery [1].

The SCDP region had a population of 363, 940 people with 132 general practices and 465 practising GPs [2]. Access to models of coordinated care was mixed across the division region, with barriers to access typically increasing with distance to major centres. The IDM study area encompassed regional communities with more limited access to specialist diabetes services compared with neighbouring metropolitan centres. The IDM program implementation focussed on the delivery of professional development for practice staff (GPs, practice nurses and practice managers); in patient management with a focus on clinical and team-based care and the identification and application of effective practice engagement strategies. These strategies included the application of an inter-professional learning framework to support team-based approaches to care combined with support to improve in practice information technology and data management, clinical up-skilling and the use of onsite diabetes educators. The success of these structural processes in improving diabetes management and health outcomes are demonstrated in previous international studies [3].

It is not the intention of this paper to report the results of the evaluation of the IDM program conducted by the SCDGP, these are available in a full report referenced in this paper. In summary, a preliminary evaluation of the program demonstrated several key improvements applicable to program design and implementation [4]. These included improved approaches to team-based and integrated care demonstrated by increased use of diabetes educators and practice nurses in diabetes care delivery; an increase in awareness and application of chronic disease management strategies and improvements in patient health outcomes demonstrated by an overall increase in the diabetic population diagnosed with an HbA1C < 7% indicating practices had maintained a clean data set. It also suggested that key elements of the clinical microsystem approach were embedded into the development, implementation and sustainability of the IDM program [4]. In order to support the further expansion of the IDM program, this study evaluated the implementation of the program against the 10 elements of a high performing clinical microsystem site [1,5].

The 4 aims of this study were to (i) determine the role of the clinical microsystem approach in triggering the successful adoption of the IDM program in general practice; (ii) determine any barriers to the implementation of the program or change adoption; (iii) determine the way a clinical macrosystem leads and supports change; and (iv) recommend the use of an applied framework to guide development and implementation of similar programs in general practice.

Ethics approval was obtained on 31st August, 2011 from the University of Queensland Behavioural & Social Sciences Ethical Review Committee (project number 2010001367).

## Methods

A qualitative evaluation approach was used and in-depth semi-structured interviews conducted with purposive sample of program staff from the SCDGP involved in the development and implementation of the IDM program and with GPs participating in the program. GP participants represented both regional and rural practices. The interviews explored perceptions of the key issues that were triggers for, or barriers to, the adoption and sustainability of the IDM program (Author: LC).

All interviews were transcribed and coded using NVivo software [6]. Codes were reviewed for duplication and clarity. A thematic analysis was undertaken and themes were reviewed for consistency against the elements of a high performing clinical microsystem, namely: (1) leadership; (2) organisational support; (3) staff focus; (4) education and training; (5) interdependence (6) patient focus (7) community and market focus (8) performance results (9) process improvement and (10) information and information technology [7,8]. Emergent themes were added to the coding framework to ensure completeness. Themes were then checked, verified and differences resolved by discussion (Authors: TJ, LC, CJ). A constant comparison method was used to improve the internal consistency of codes. Negative cases were identified and included in the results and these were defined as themes that were not present in the 10 elements of high functioning clinical microsystems.

## Results

Ten key informant interviews were completed, 5 with SCDGP key informants involved in the design, implementation and internal evaluation of the IDM program and 5 with GPs who participated in the IDM program from individual practices. One of the GPs was also directly involved in the design and delivery of the inter-professional learning-based workshop sessions (Table 1).

**Table 1:** Interviewees’ positions and roles in IDM program

**Position**	**Role in program**
**Program facilitator**	Background review and development of program funding application (processes and key performance indicators)
**Team leader**	Implementation and monitoring; engagement of practice staff
**Evaluator**	Process monitoring and feedback; outcome evaluation
**Diabetes educator**	Clinical support to participating practices; engagement of clinical staff
**Information management and technology**	Engagement of practices; application of the clinical audit tool; database cleansing; performance feedback
**GP 1**	Education committee member (inter professional development)
**GP 2**	Participant (second phase)
**GP 3**	Participant
**GP 4**	Clinical fellow
**GP 5**	Participant (second phase)

**Figure 1:**
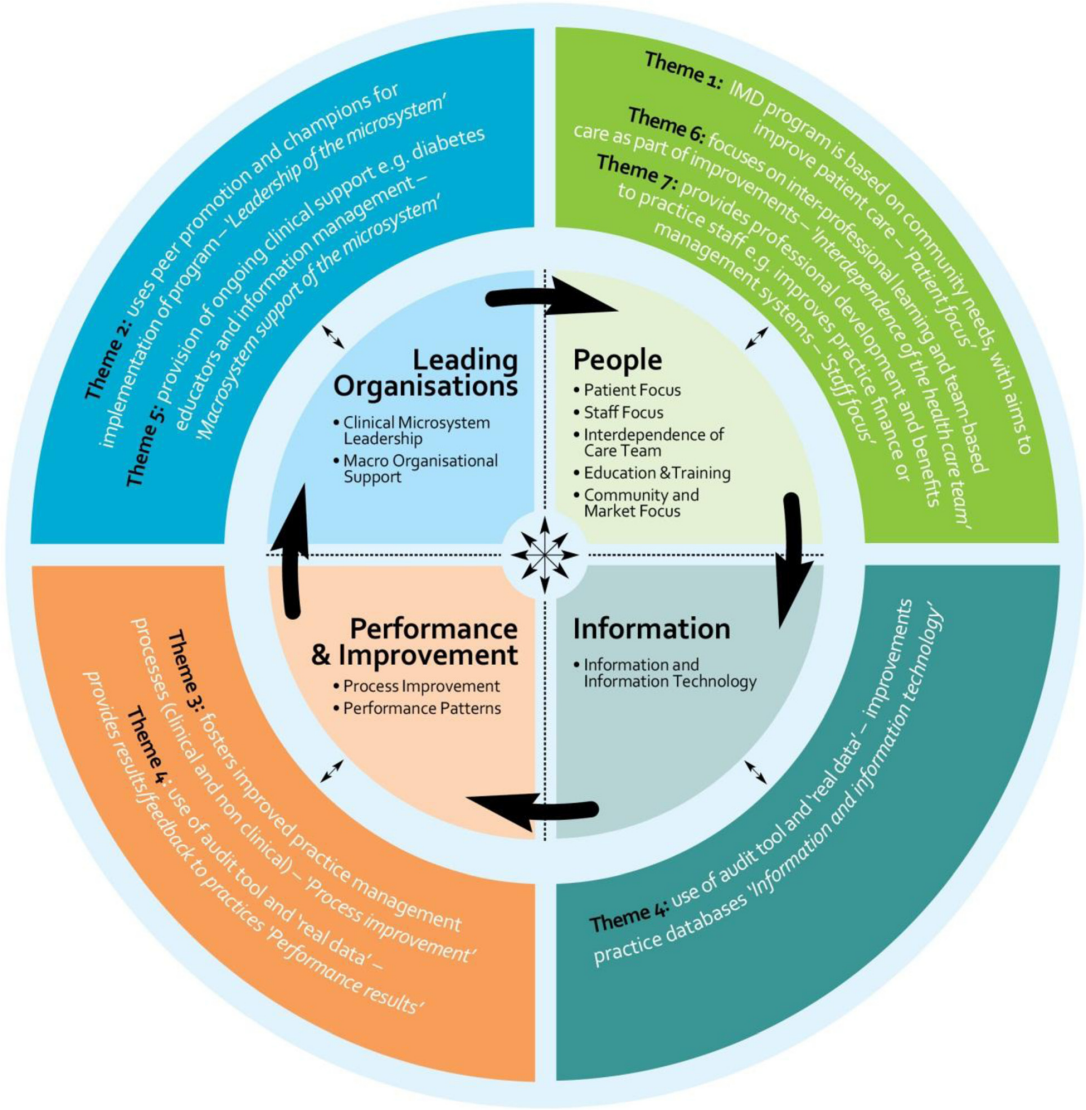
**Characteristics of high performing clinical microsystem sites as identified by Nelson et al. 2007**[7]**.**

Seven key themes emerged which were identified by participants as key triggers for change and the successful adoption of the IDM program. Figure 1 illustrates the themes (listed on the outside of the circle) against the elements of a high performing clinical microsystem (contained in the centre of the circle).

### Theme 1: Factors that motivated participation in the IDM program

Interviewees noted that the IDM program was based on previous research conducted by the division. This research sought to identify key areas for improved health care delivery as perceived by health professionals and local consumer representatives. Following this, program staff determined the factors specific to each practice that motivated practice staff to engage in the IDM program. These motivating factors varied between practices but generally encompassed one or more of a combination of the following 3 areas: (1) a desire to improve patient health outcomes; (2) a desire for the potential financial benefits or (3) improvements in time management and the working processes of the practice.

Time, money and patient outcomes as the key motivators for GPs … because each practice it sort of was considered ‘let’s try seeing which of those things pushes the buttons for the practices’. So we really, I think, got a bit more clued in, in terms of ‘OK, for this practice it’s clear from the comments they made that time is the button… for this one it’s money, for this one its patient outcomes and they don’t give a rats about financial implications they just want the best possible thing… (Staff 4)

My diabetic patients are managed better than they were … I certainly know I am much more confident in what I do, and the outcomes are probably best measured by HbA1C’s so far have dropped down to sort of more appropriate levels.. But if there can be financial gain as well as gain for your patients, the well-being of your patients, that’s the incentive (GP 3)

### Theme 2: Champions and leaders

Interviewees perceived benefits of using both internal champions (those people working within the participating practices) and external champions (peers or ‘experts’, from outside the participating practices) in the introduction and ongoing implementation of the program. The use of these champions ensured the ongoing motivation of the health care professionals, while the external champions, most particularly those presentations provided by other general practitioners, demonstrated the benefits of the program in the broader regional setting. Interviewees felt that the key champion in most practices was the practice manager and their commitment to the program and particularly models of inter-professional care and ‘team-work’ ensured long term success.

The other focus was using other GPs who were running clinics and had pretty good chronic disease systems in place within their own practices, so bringing them along to speak to the GPs, that was a big plus … That approach was quite instrumental in changing some GP views (Staff 1)

### Theme 3: Focus on process improvement

Interviewees perceived that an important feature of the program and key trigger for change was the focus on process improvement and the ability to change implementation practices throughout the study.

And we did have to change our approach. So with our first wave of 6 practices and our second wave of practices, we tweaked and modified and recruited differently (Staff 1)

I do feel there was quite a difference between wave 1 and wave 2. With wave 1 I think we went in there just expecting the full package to just happen. We expected to get the clinic up and running… With wave 2, because they’d had a lot of exposure to diabetes education and seminars and workshops that had been provided… they were actually at the point that perhaps wave 1 were almost towards the end of their program… so we knew that (Staff 3)

### Theme 4: Use of audit tool and ‘real data’

The use of the audit tool and the ability for the practice staff to see ‘real data’ relating to their practice patients provided another significant trigger in the uptake of the IDM program. In addition, the SCDGP support staff were able to provide participating practices with evidence including accurate diabetic patient numbers and an ability to see improvements in the rates of identification and screening of diabetes.

Showing them real data, I think that was the turning point… showing that this is what you can achieve. The outcomes and the patent data was the most effective (Staff 5)

And secondly, to feel that these programs are going to benefit both general practice, and that includes doctors and nurses in general practice, and probably most importantly to benefit patients and the positive here is really that I think that the outcomes are going to be able to be seen to be positive as a result of the work that’s gone into the program (GP 3)

### Theme 5: Provision of clinical support

Most interviewees perceived that the provision of practical support to the practices as part of program implementation was a key feature that lead to successful practice engagement and change in general practice. The most notable source of onsite support was related to the time and clinical expertise provided by the onsite diabetes educators in relation to patient management. Having learnt from the success of the onsite information management support, the same onsite support was then initiated with the diabetes educators in order to further facilitate changes in patient management.

… the enabler was giving them the diabetes educator’s time … They didn’t have to pay for that and they were getting the benefit of being able to ask and getting advice and clinical advice. They key benefit of having that person on the ground was that they were able to work with the nurse in a mentoring capacity (Staff 2)

### Theme 6: Supportive relationships

The development of strong supportive relationships was a significant factor in the uptake of the IDM program. The most important relationships were those established between the participating practice staff, chronic disease specialists and diabetes educators as well as the rapport they built with the information managers.

*I think they* [the GPs] *really appreciated the support. I know with one particular GP because of the inter-professional learning sessions that we provided and with me going into the practice not long after that session, she really engaged me as well as the practice nurse in consultation with the patient. She could then consolidate her knowledge around insulin initiation for example (Staff 3)*

It was also the level of support that they were given and the rapport that they’d built up with the IT people and with their diabetes educator (Staff 1)

### Theme 7: Education and training

The education and training component of the program was perceived as an important characteristic, in particular the structure and delivery of the inter-professional training sessions. Two interviewees noted that making attendance at the workshops a mandatory part of program participation was the key to fostering team-based care amongst the participating health care professionals.

*They* [the GPs and staff] *had to attend the compulsory workshops as part of the contractual arrangement, and part of the agreement was that a GP had to attend, so what happened was they all came along and then the ideas started to flow… (Staff 2)*

The incentive was extra training and the course that was actually funded for me, so the incentive of actually getting some up-skilling and some recognised clinical skilling, at no cost to myself, was certainly an incentive. And having done that, the incentive now is just to keep that knowledge there and increase it further and use it. Just helps in my enjoyment of medicine as a whole (GP 1)

Figure 1 demonstrates how characteristics of the IDM program, as identified in the interviews, related to those characteristics of high performing clinical microsystem sites identified by Nelson et al. 2007 [7]. The outer layer of the circle includes characteristics of the IDM program as identified by informants and relates these to the characteristics of high performing clinical microsystems.

## Discussion

The interplay of these characteristics is complex and interrelated. For example, the incorporation of strong information technology approaches resulted in the ability for the division to foster improved management processes and provide direct feedback on the performance of each practice as the program continued. The IDM program had a strong patient focus which resulted from an identified community need. In addition, improved patient care and health outcomes, fed back to practices as part of the IDM program implementation, were strong motivators for GP participation. The identification of GP peer support, particularly the use of a GP to present the positive outcomes of improved diabetes management, was used during education and training sessions to underline the practical benefits of the program and evidence of its effectiveness in general practice settings. The division provided onsite diabetes educators to provide ongoing clinical support to GPs and nurses involved in the program. This resulted in the opportunity for onsite learning and the development of new clinical and management skills as part of normal practice. The use of the inter-professional learning framework to guide the practice workshop sessions promoted team-based care and facilitated the adoption of new roles and responsibilities by staff.

The division developed a formal implementation program however much was learnt from the first stages of the program to the final stages. Program strategies were adapted and changed during the course of program delivery. This flexibility allowed the SCDGP to tailor their approaches to better meet the needs of each practice in the second phase of the program. Overall, the division employed and led a clear and simple approach to the implementation of the program in each practice and this was seen as a crucial trigger for change.

Finally, the application of the audit tool and the improvements to patient data management enabled improvements to patient management and health outcomes (as measured by improved screening rates and lowering HbA1C results and presented in the SCDGP evaluation reports) to be evidenced in each practice. Ongoing feedback of these improvements, as part of a formal evaluation process, was made to each general practice. This enabled the division to continually adjust the strategies to ensure that positive outcomes were achieved and practices to clearly see the benefits of the program to their own patients.

While all aspects of the clinical microsystem approach appeared effective in the design, implementation and adoption of the IDM program, several characteristics were crucial. The identification of champions of change in both the division and participating practices, the celebration of positive achievements resulting from practice change and the use ‘real data’ from practices, to demonstrate improved health outcomes for patients from the practice were instrumental in motivating participating GPs to both implement and sustain changes in their diabetes care delivery. The education and training strategies employed by the division were simple and flexible. They encompassed both formal workshops and onsite education and training as part of practise. The opportunities for GPs to attend workshop sessions which were clinically relevant and not time-consuming was an important feature of the successful uptake of the program. Informal onsite education and training enabled GPs to continue to up-skill in diabetes care as part of everyday practise.

### Limitations of the study

This work aimed to explore the feasibility of the clinical microsystem approach as a valid process for informing the development and implementation of the program from the perspective of the staff involved.

There are two limitations to this study, namely: (1) characteristics, such as the divisions of general practice, that are unique to the Australian primary health care context; and (2) the small numbers of staff interviewed. Following this, the findings from these interviews may not be directly transferable across other Australian or international settings.

## Conclusion

In designing and redesigning health care, the clinical microsystems approach offers a pathway for the effective uptake of innovation in primary health care delivery in Australia; it offers a way to integrate structure, process and outcomes of care [5]. It also offers a framework for reviewing improvements in the health care delivery process and corresponding improvements in patient health outcomes. The approach may be relevant to initiating and sustaining a change in relation to a range of complex and chronic diseases which involve a wide range of service delivery providers in primary health care. Therefore clinical microsystem approach should be considered in the development and implementation of primary health programs and can be used by macro-organisations seeking to initiate and support change in the primary health care sector.

## Competing interests

The authors declare that they have no competing interests.

## Authors’ contributions

All mentioned authors made substantial contribution to this research and manuscript: TJ was involved in research protocol development, submission of ethics application, and different phases of the data collection and analysis, and writing of manuscript. LC was involved in all phases of data collection and analysis, and writing of manuscript. CJ participated in the design of the study, provided important intellectual content and was involved in the drafting of the manuscript. JM was involved in recruiting of participants and data collection. All authors read and approved the final manuscript.

## Pre-publication history

The pre-publication history for this paper can be accessed here:

http://www.biomedcentral.com/1471-2296/15/32/prepub
